# Analyzing tiger interaction and home range shifts using a time-geographic approach

**DOI:** 10.1186/s40462-024-00454-0

**Published:** 2024-02-03

**Authors:** Yifei Liu, Somayeh Dodge, Achara Simcharoen, Sean C. Ahearn, James L. D. Smith

**Affiliations:** 1 Department of Geography, University of California, Santa Barbara, USA; 2 Protected Area Administration, Office 12, Department of National Parks, Wildlife and Plant Conservation, Nakhon Sawan, Thailand; 3 Hunter College - CUNY, New York City, USA; 4 Department of Fisheries, Wildlife and Conservation Biology, University of Minnesota, Twin Cities, USA

**Keywords:** Time geography, Tiger movement, Interaction analysis, Interaction duration

## Abstract

**Background:**

Interaction through movement can be used as a marker to understand and model interspecific and intraspecific species dynamics, and the collective behavior of animals sharing the same space. This research leverages the time-geography framework, commonly used in human movement research, to explore the dynamic patterns of interaction between Indochinese tigers (*Panthera tigris corbeti*) in the western forest complex (WEFCOM) in Thailand.

**Methods:**

We propose and assess ORTEGA, a time-geographic interaction analysis method, to trace spatio-temporal interactions patterns and home range shifts among tigers. Using unique GPS tracking data of tigers in WEFCOM collected over multiple years, concurrent and delayed interaction patterns of tigers are investigated. The outcomes are compared for intraspecific tiger interaction across different genders, relationships, and life stages. Additionally, the performance of ORTEGA is compared to a commonly used proximity-based approach.

**Results:**

Among the 67 tracked tigers, 42 show concurrent interactions at shared boundaries. Further investigation of five tigers with overlapping home ranges (two adult females, a male, and two young male tigers) suggests that the mother tiger and her two young mostly stay together before their dispersal but interact less post-dispersal. The male tiger increases encounters with the mother tiger while her young shift their home ranges. On another timeline, the neighbor female tiger mostly avoids the mother tiger. Through these home range dynamics and interaction patterns, we identify four types of interaction among these tigers: following, encounter, latency, and avoidance. Compared to the proximity-based approach, ORTEGA demonstrates better detects concurrent mother–young interactions during pre-dispersal, while the proximity-based approach misses many interactions among the dyads. With larger spatial buffers and temporal windows, the proximity-based approach detects more encounters but may overestimate the duration of interaction.

**Conclusions:**

This research demonstrates the applicability and merits of ORTEGA as a time-geographic based approach to animal movement interaction analysis. We show time geography can develop valuable, data-driven insights about animal behavior and interactions. ORTEGA effectively traces frequent encounters and temporally delayed interactions between animals, without relying on specific spatial and temporal buffers. Future research should integrate contextual and behavioral information to better identify and characterize the nature of species interaction.

## Background

### Study goals

Interspecific and intraspecific interactions play essential roles in shaping species collective dynamics in ecosystems [1–3]. They are key drivers in forming behavioral patterns and inter-individual dynamics, such as intraspecific interactions, predator–prey relationships [4–6], sexual selection [7, 8], parasitism [9, 10], and mutualism [11, 12]. The collective dynamic interactions between animals drive population and community evolution [13–15]. Moreover, predation and competition are considered as main factors influencing carnivore resource selection and population dynamics [16–18]. As such, spatial and temporal attraction and avoidance are key mechanisms governing interspecific social dynamics [19].

Tiger, as a top predator in many Asian ecosystems, is a keystone species and serves as a flagship species for conservation in the region [20, 21]. Like most felids, the tiger is intra-sexually territorial [22, 23]. They interact through chemical and vocal communications. Adult tigers scent mark trees to alert other tigers of territorial boundaries and to avoid direct encounters that may result in serious injuries or even mortality to the loser [24]. The scent marks seem to last up to 3 weeks according to field observations [24]. Female tigers’ scent marks also reveal their reproductive status to male tigers of their approaching estrus [25] and thus serves as a temporally delayed interaction between tigers. Breeding female tigers occupy defended home ranges (territories), and raise their offspring solely by hunting prey within their home ranges [23, 26]. Thus, the home range size of a female tiger is a function of prey density [26–28]. Subadult tigers usually disperse from their mother’s territory within 2 months of their mother giving birth to subsequent litters [29]. Female tigers can settle partially within or adjacent to their mother’s territory or they disperse, while male tigers are expelled from their father’s territory [29, 30].

In this paper, we propose and assess the applicability and performance of an object-oriented time-geographic analytical method for movement interaction analysis, named ORTEGA [31] in tracing and understanding tiger intraspecific interaction behavior and home range shifts through their movement in space and time. Using a unique data set of long term tracking data of tigers collected at a 1-h sampling rate in Thailand’s Western Forest Complex (WEFCOM), this study follows several objectives: First, we demonstrate ORTEGA’s application to quantify spatio-temporal interaction patterns within a large network of tigers (14 years of tracking data of 67 tigers including total of 285,648 GPS observations). Second, focusing on dyadic interaction patterns among five tigers of different genders, relationships, and life stages with connected home ranges between September 2018 and August 2020 (total of 40,860 GPS observations), we analyze concurrent and delayed interaction patterns among these tigers and investigate how their collective dynamics associate with changes in their home ranges. Finally, in a comparative experiment, we assess the outcomes against the commonly used proximity-based interaction analysis techniques [32]. The study area, WEFCOM (19,000 km^2^), holds the largest population of the Indochinese tiger (*Panthera tigris corbeti*) [33]. Because this population is the only potential source population for the recovery of this subspecies, understanding the underlying processes that drive its ecology and interaction behavior is important for the conservation of tigers in Southeast Asia. Analyzing tiger interaction and movement patterns can provide needed information for not only restoration, but the expansion of tiger habitat in the region.

### Movement interaction analysis

Dyadic interaction between animals can be static or dynamic [32, 34]. Static interaction is often quantified as the spatial overlap of the activity spaces of two animals sharing the same geographical space but not necessarily moving at the same time. Dynamic interaction occurs when two animals move in close proximity over a certain time interval [35, 36]. Dynamic interaction can be classified into encounters, concurrent, and delayed interaction, based on the duration and the lag of interaction [31, 37]. A concurrent interaction occurs when individuals move synchronously in space and time. If the concurrent interaction lasts only for a short period of time (e.g. a few minutes), it can be considered as an encounter [37]. A delayed interaction happens when individuals visit the same location asynchronously with a time lag [38].

In movement ecology, a range of metrics have been developed to quantify dyadic dynamic interactions using animal tracking data: the proximity index [34, 39], the coefficient of sociality [40], the coefficient of association [41], the half-weight association index [42], the coefficient of interaction [43, 44], the cross sampled entropy [45], the correlation indices [46, 47], and the dynamic interaction index [48]. Most of these measures rely on the spatial proximity between two entities and require user-defined spatial and temporal thresholds [32, 49, 50]. Although proximity-based approaches are often applied in detecting interaction when entities are tracked simultaneously in time, they are limited in identifying interaction that happens non-synchronously or is not captured in non-synchronous tracking [37].

### Time geography and movement analytics

**Fig. 1:**
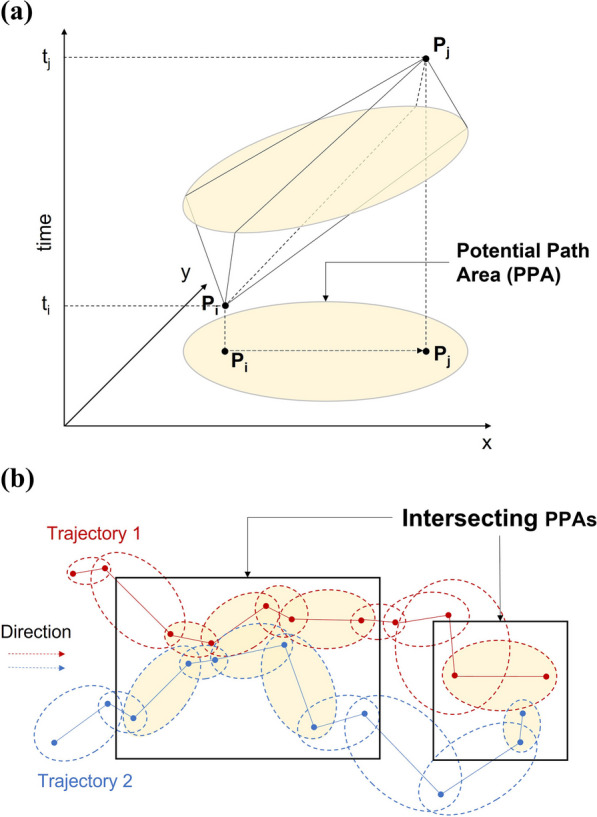
Illustration of **a** the space–time prism in a three-dimensional space–time cube and its projection on the 2D geographic space, known as the potential path area (PPA); **b** PPA intersections (light yellow ellipses) to identify potential interaction between two moving entities (modified from [31, 37]). Two trajectories are outlined in red and blue. Two sets of continuous intersection segments are outlined using black rectangles, capturing a concurrent interaction on the left, and an encounter segment on the right

More recent approaches to movement interaction analysis incorporate the time geography framework [51] to consider uncertainty and gap in movement tracking data [52]. The time geography framework originates from human mobility research [51]. It models the accessible locations to an entity (e.g. an animal) moving between two fixed locations given a time budget to travel between the two locations and a maximum speed with a three dimensional space–time prism (Fig. 1a). The projection of this prism in the geographic space is an ellipse called the Potential Path Area (PPA), which has been widely used to study human activity space and movement patterns. The PPA delimits the locations that can be reached by the individual given a time budget and the maximum speed capacity of the individual [53, 54].

The time-geographic PPA is comparable to the Brownian Bridges [55] in a sense that both models compute potential areas between two recorded locations where the individual can be observed. While the Brownian Bridge, which is often represented as a raster probability surface, estimates the probability of the individual being at different locations, the PPA delimits the maximum area that can be reached by the individual during a time window and at a certain speed. Compared to the Brownian Bridge, the time-geography uses a PPA ellipse (represented as a vector polygon) with the same visit probability across its surface to model activity space. To make time-geographic probabilistic, a Brownian Bridge [56] or a random walk model [57] can be integrated in the PPA computation and to generate a visit probability surface.

Leveraging time geography, the recent time interaction analysis methods identify potential areas for interaction when the PPA ellipses of different individuals overlap along their trajectories. Potential interactions between trajectories can be identified via intersecting PPAs, as illustrated schematically in Fig. 1b, with a user-defined time-distance window [52, 58], or between two subsequent tracking points with a more flexible tracing capacity over time [31, 38].

Beside modeling human movement, time geography has also been used in wildlife home range and interaction analysis [31, 52, 58–62]. Down et al. [61, 62] proposed a voxel-based approach to quantify the probability of physical interaction within the space–time prism for multiple individuals. This approach is analogous to transferring the two dimensional Brownian Bridges to a three dimensional space–time cube. Hoover et al. [58] extended the joint potential path area (jPPA) method [52] to identify the temporally asynchronous-joint potential path areas (ta-jPPA) that map potential locations for delayed dyadic interaction with a user-defined time increment. Using a similar approach, Dodge et al. [31] introduced an Object-oRiented Time-Geographic Analytical approach (ORTEGA) to extract concurrent and delayed interaction patterns in both human and animal tracking data. In contrast to the previous time geographic approaches, ORTEGA is more efficient as it models PPAs as objects which have attributes and behaviors to optimize the tracing of PPA intersections spatially and temporally in larger moving object data sets [31]. Hence, ORTEGA is capable of analyzing interaction among dyads as well as in animal networks with more than two individuals. This study harnesses ORTEGA and its extensions [31, 38] to identify and trace concurrent and delayed interaction patterns among multiple tigers. The detailed methodology is described in “Interaction detection and duration computation using ORTEGA” section.

## Methods

### Interaction detection and duration computation using ORTEGA

The methodology of this study consists of two processes: (1) detection of interaction, and (2) computation of duration of interaction.

The time geographic approach, ORTEGA [31], introduced in “Time geography and movement analytics” section, is applied to identify potential interactions between two moving entities along their trajectories. That is, after pre-processing of GPS tracking data, PPA arrays are computed as ellipses. Each ellipse is generated using consecutive pairs of GPS points along its long axis, factoring in the time interval (i.e. the time budget to travel between the two points) and a maximum speed.

The maximum speed, denoted as $v_{max}$, is calculated using an Exponential Weighted Moving Average (EWMA) method [63, 64], as detailed in Eq. 1 [31].

1 $$\begin{aligned}&s_i = {\left\{ \begin{array}{ll} v_{i} &{}\quad {\text{if }} \lambda = 1 \\ \sum \limits _{k=0}^{n-1}\lambda (1-\lambda )^{k} v_{i-k} &{} \quad {\text{if }} 0<\lambda <1\\ \end{array}\right. }\\&v_{max} = \epsilon s_{i} \end{aligned}$$

Here, $s_i$ represents the smoothed speed at the current point *i*, and $\lambda$ is the smoothing constant. The use of $\lambda$ in the EWMA serves as a decay factor, effectively controlling the influence of past speed data points $v_{i-k}$ on the current speed estimation. Specifically, when $\lambda$ is set to 1, the current point’s speed $v_i$ is given the full weight to the current speed. However, as $\lambda$ approaches 0, the formula progressively incorporates a broader range of historical speed data, thereby enhancing the smoothing effect. This is reflected in the summation term, which weights past speeds with exponentially decreasing significance based on their location *k* within the number of previous data points *n*. The error term $\epsilon$, set as 1.25 in this study, provides additional flexibility. This adjustment allows the maximum speed calculation to deviate by up to 25% from the smoothed average, accommodating the natural variability and unpredictability of animal movements.

Very large PPAs that were generated due to data gaps (i.e. larger than three times the standard deviation of the sampling intervals) were removed from the data set to avoid erroneous intersection [47]. To accelerate computational speed, a Compressed K-Dimensional tree (CKD-tree) method is applied based on the centroid points of the PPAs to filter the PPAs within certain spatial and temporal intervals for interaction analysis. This way, the dyads that don’t share a tracking timeline or are not proximate geographically can be excluded in interaction analysis. A potential dyadic concurrent interaction is detected if the PPAs of two animals are intersected in time, while the delayed interactions are extracted using a time lag (i.e., the time difference between the starting time of two spatially intersecting PPAs) [31].

The continuous subsequences of PPA intersections are traced to quantify the potential interaction sequences and the duration of concurrent interaction [38]. Duration of interaction is computed by the difference between the end time (maximum time) and the start time (minimum time) of the continuous intersection segments. This duration is used to distinguish between the brief encounters, either intentionally or accidentally, between the tigers versus the longer intentional concurrent interaction of tigers moving together for a period of time. Detailed interaction analysis algorithms and ORTEGA workflow are presented in “Appendix” (Fig. 17). Readers are referred to [65] (in review) for more technical details, Python source codes, and a step-by-step tutorial on how to apply ORTEGA for interaction analysis.

### Study area and data

The core study area is the Huai Kha Khaeng Wildlife Sanctuary, one of 17 protected areas that make up WEFCOM [66]. The data set includes a total of 67 tigers within this sanctuary which were captured, immobilized (UMN IACUC protocol 2204-39926A), and fitted with GPS collars (VECTRONIC Aerospace GmbH) [33]. The tigers’ movement were tracked at 1 h sampling rate from 2009 to 2022, amounting to 285,648 tracking points. The tracking duration for each tiger varies, spanning from a few months to nearly 3 years.

**Fig. 2:**
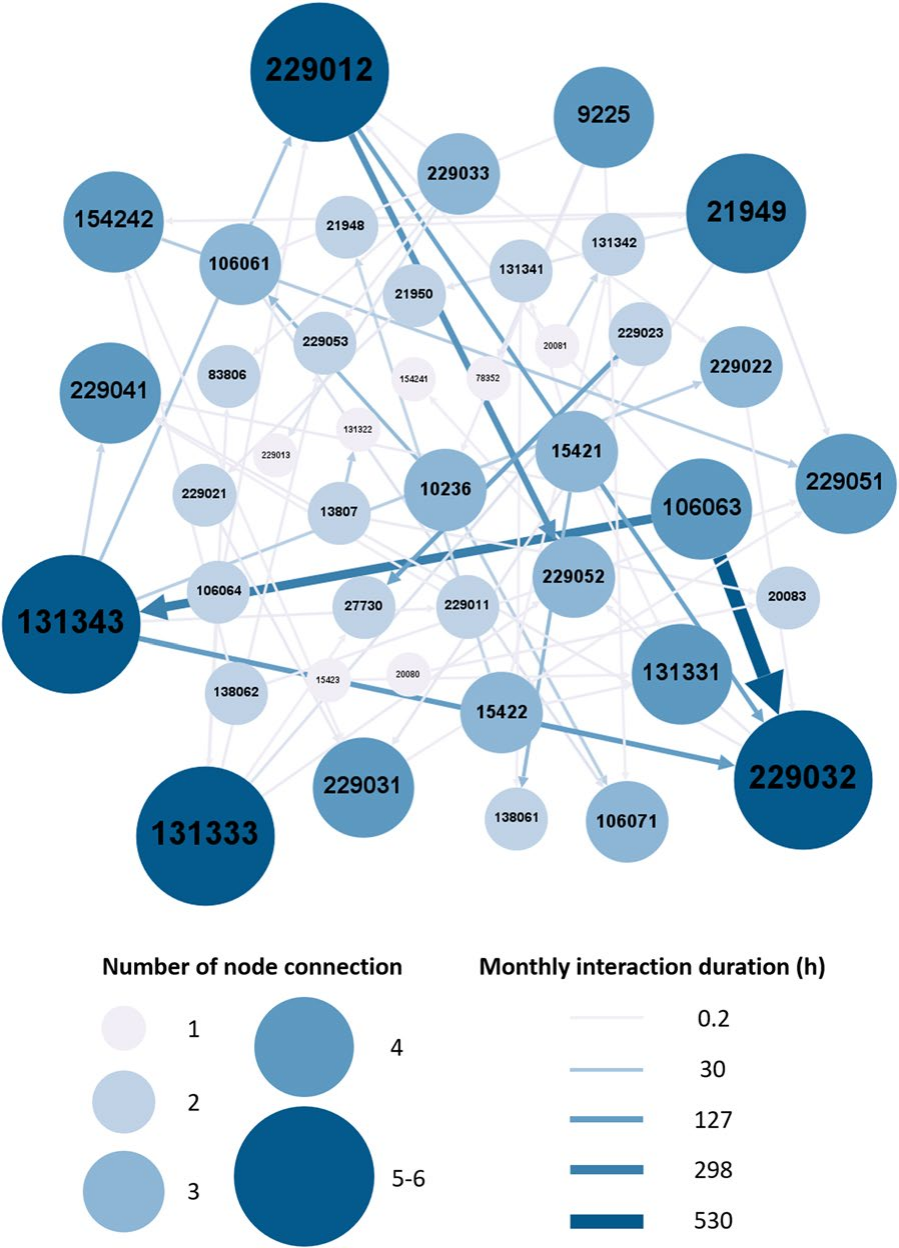
Illustration of the interaction network of 42 tigers. The size of each node in the network is proportionate to the number of concurrent interactions the tiger has with other individuals. The node in darker blue represents more connections (mean of 2.8 ± 1.5 connection, maximum 6 connections for the largest node). The edges that are displayed in a darker blue and with thicker lines represent a higher frequency of concurrent interactions between the dyads (mean of 28 ± 82.3 h per month, maximum 530.1 h per month for the thickest edge)

Using ORETGA, from the 67 tigers in the data set, 42 display concurrent interactions. It is important to note that not all tigers share home range boundaries or are tracked during the same time period. Hence, ORTEGA only captures interaction among tigers with shared boundaries and timelines. The interaction network of these 42 tigers is visualized in Fig. 2 using Gephi [67]. This visualization represents the total duration of interaction during the tracking period, which varies from a minimum of 0.15 h per month to a maximum 530.1 h per month. The nodes in the network correspond to individual tigers, identified by their respective IDs. The larger nodes shown in a darker blue color (IDs: 131343, 229012, 229032, and 131333) capture tigers with a higher frequency of interactions with other tigers during the tracking period. The edges represent the number of interactions between tiger dyads, with darker blue and thicker lines capturing more interactions. This network indicates that most of these 42 tigers have few concurrent encounters (average of $28.2 \pm 82.3$ h per month) and mostly stay away from each other.

**Fig. 3:**
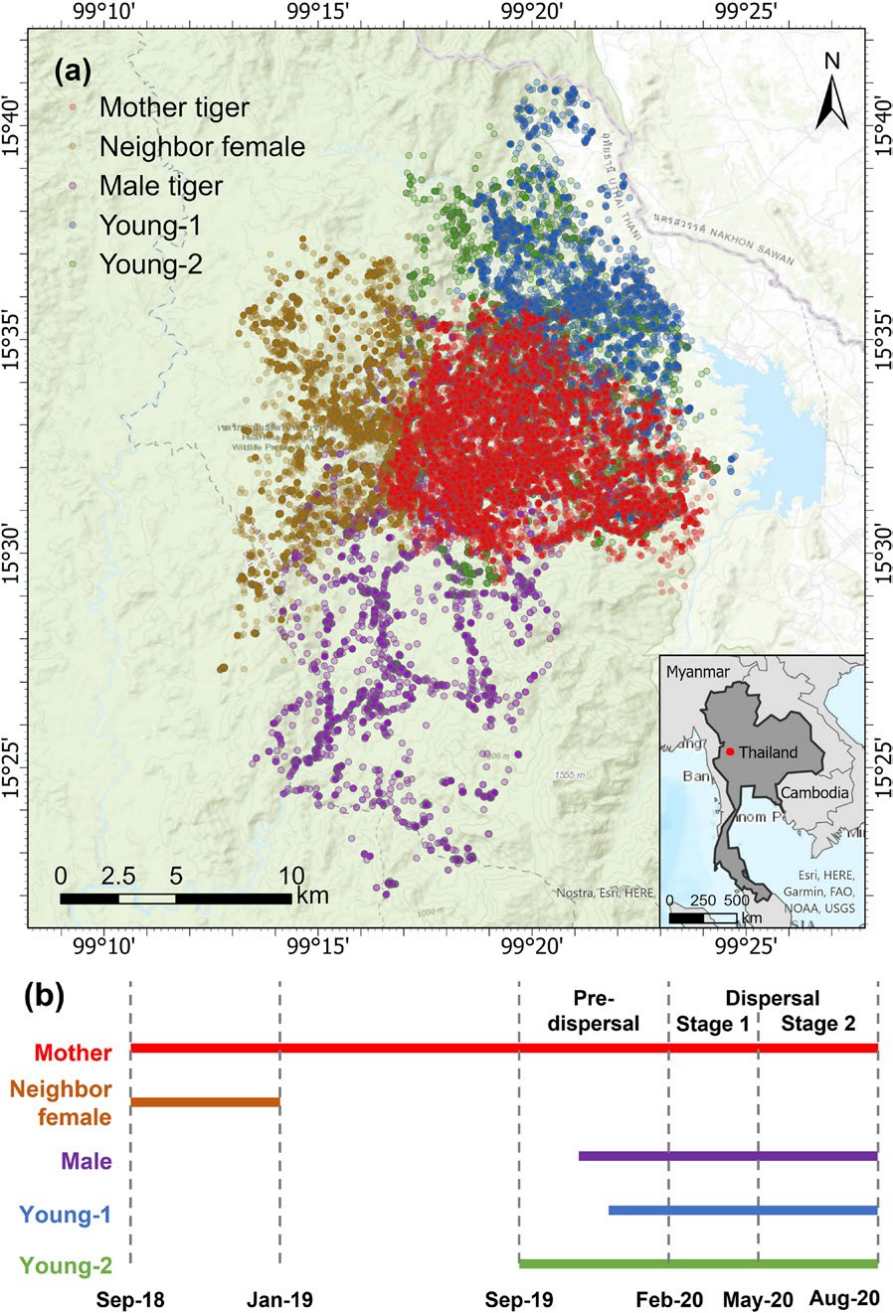
**a** Study area and GPS tracking data of mother tiger (in red), young-1 (in blue), and young-2 (in green), neighbor female (in brown), and male tiger (in purple). **b** The tracking timelines of the five tigers in their representative color. Except for the neighbor female that is tracked on a different timeline, the timeline of the other four tigers are divided into three stages: pre-dispersal, dispersal Stage 1, and dispersal Stage 2

Among the network, five tigers with overlapping home ranges and specific relationship dynamics exhibit a higher rate and/or interesting patterns of interactions. Therefore, in what follows, the study focuses on a detailed analysis of tracking data of these five tigers (see in “Results” section), including: a female tiger (ID: 131343, named “mother tiger” herein, 16,899 points) and her two male young, age 1–2 years old, (ID: 229012, named “young-1”, 4587 points; and ID: 229032, named “young-2”, 8129 points); and two other adult tigers with adjacent or shared portions of home ranges with the “mother tiger”: one female (ID: 229011, named “neighbor female”, 4819 points), one male tiger (ID: 229022, named “male tiger”, 6426 points). Figure 3 maps this data set and the study area. The tigers were tracked between September 2018 and August 2020 (total of 40,860 GPS observations), but not all at the same time, as illustrated in the tracking timelines shown in Fig. 3b.

**Fig. 4:**
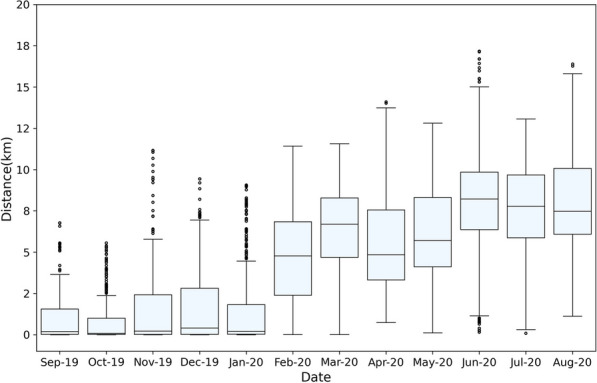
Monthly distance distribution between mother and young-2 tiger. The increased distance between mother and young after January 2020 shows that the young is beginning to shift out of his natal area

To analyze tiger intraspecific interaction between different genders and ages, we consider the following dyadic relationships: mother–young, female–female, female–male, and male–male. For each dyad, the tracking data during the maximum common duration available are used (Fig. 3b). That is, the mother tiger and the neighbor female tiger interactions are analyzed from September 2018 through January 2019, while the interactions between the mother tiger and her young are analyzed during September 2019 and August 2020. The tracking of young-1 started in December 2019, right before the young become semi-independent. The interactions between the male tiger and the mother tiger are analyzed from November 2019 through August 2020. To characterize the interaction patterns among the young and the other tigers, we consider three stages as follows (Fig. 3b): (1) Pre-dispersal: before the young disperse (September 2019–January 2020), (2) Dispersal Stage 1: when young expand their home range beyond the boundary of their mother’s territory (February 2020–April 2020), (3) Dispersal Stage 2: after the male’s week-long visit with the female (May 2020–August 2020). These phases are characterized and identified based on the spatial distributions of their tracking points, the point-to-point distances between the animals (see Fig. 4), changes in spatial overlap with their mother’s home range, and the continued shift out of their mother’s territory after she associates with a new resident male.

In order to understand how the home ranges of the five tigers overlapped spatially, we apply the convex hull of 95% tracking points of five tigers to represent the “used” home range [68]. The home range area for each tiger and the overlapped home range proportion for each dyad are presented in Tables 1, 2, 3, and 4.

**Table 1:** Summary of overlapped home range proportion (%) of mother tiger, male tiger, young-1, and young-2 at three stages

Dyad	Pre-dispersal	Dispersal	
		Stage 1	Stage 2
Mother–young-1	99.6	35.2	10.1
Mother–young-2	99.5	61.0	26.2
Male–young-1	19.2	0.0	11.9
Male–young-2	32.1	15.5	29.4
Male–mother	35.0	40.5	83.2

For each *dyad*(*x*, *y*), the overlapped area of *x* and *y* is divided by the home range area of *y*

**Table 2:** Summary of overlapped home range areas (km^2^) of mother tiger and neighbor female tiger in 5 months

Tiger	Sept-18	Oct-18	Nov-18	Dec-18	Jan-19
Mother–neighbor female	0.0	0.6	3.8	3.9	2.3

## Results

This section describes the results of our interaction analysis, organized based on different tiger dyads. Since the time interval of the tracking data is 1 h, the time lag of two spatially intersected PPAs equal to or shorter than 1 h is considered as a concurrent interaction, while the time lag longer than 1 h is regarded as a delayed interaction.

### Duration of concurrent interaction

**Fig. 5:**
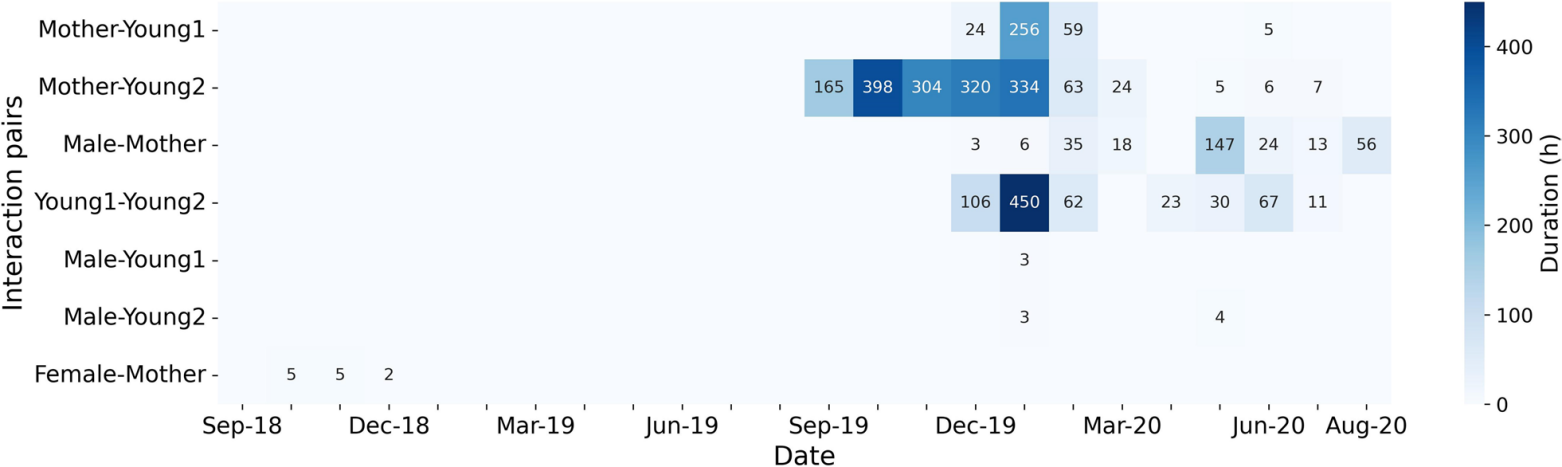
The heat map of monthly duration of concurrent interactions among the five tigers in each month. Darker colors represent longer duration of monthly concurrent interactions for each dyad

The duration of monthly concurrent interactions (time lag $\le 1$ h) between the five tigers captured in the data are summarized in Fig. 5. The outcomes represent how long tigers potentially interacted (i.e. quantified as accumulative duration of continuous PPA intersection sequences) each month. In general, the results show only a few instances of relatively short concurrent intra-sexual interactions between adults. Total duration of monthly concurrent interaction between the adult male and the young tigers is less than 4 h. The neighbor female and the mother tiger only interact for a brief period (average 4 h) during the tracking period of September through December 2018.

Most of the mother–young concurrent interactions are detected during the pre-dispersal period (September 2019–January 2020, average monthly duration of $257.0 \pm 116.2$ h), peaking in October 2019. However, the concurrent interaction detected in dispersal Stage 1 (February 2020–April 2020) between the mother and the young is significantly shorter (average monthly of $24.3 \pm 27.3$ h), and even becomes less in dispersal Stage 2 (May 2020–August 2020, monthly average of $2.9 \pm 2.9$ h).

Interestingly, as the duration of mother–young interaction decreases, the male–mother interaction increases. The male tiger and the mother interact concurrently for a total of 147 h in May 2020, and then the duration drops to an average of 31 h per month in the following months. The two young tigers show a notable interaction in January 2020, interacting for approximately 450 h, which appears to mark the onset of dispersal. Afterward, Stage 2 of dispersal starts in May 2020 when the male associates with the female from May 22 to May 29, 2020, as described below (Table 6).

### Comparison between ORTEGA and proximity-based approach

A comparative analysis of ORTEGA and the existing proximity-based approach is conducted, exploring the impact of varying time lag parameter and spatial buffer (a threshold to define the close contact between two individuals) when quantifying the duration of potential concurrent interactions. As an illustrative example, we employ the tracking data of the mother tiger and the young-2 tiger, specifically during pre-dispersal, dispersal Stage 1, and dispersal Stage 2. Because of the lack of ground truth data, we assume that a greater number of interactions occur in the pre-dispersal stage when the mother tiger and her young stay together, and fewer interactions occur during the dispersal stages when the young become independent of their mother.

**Fig. 6:**
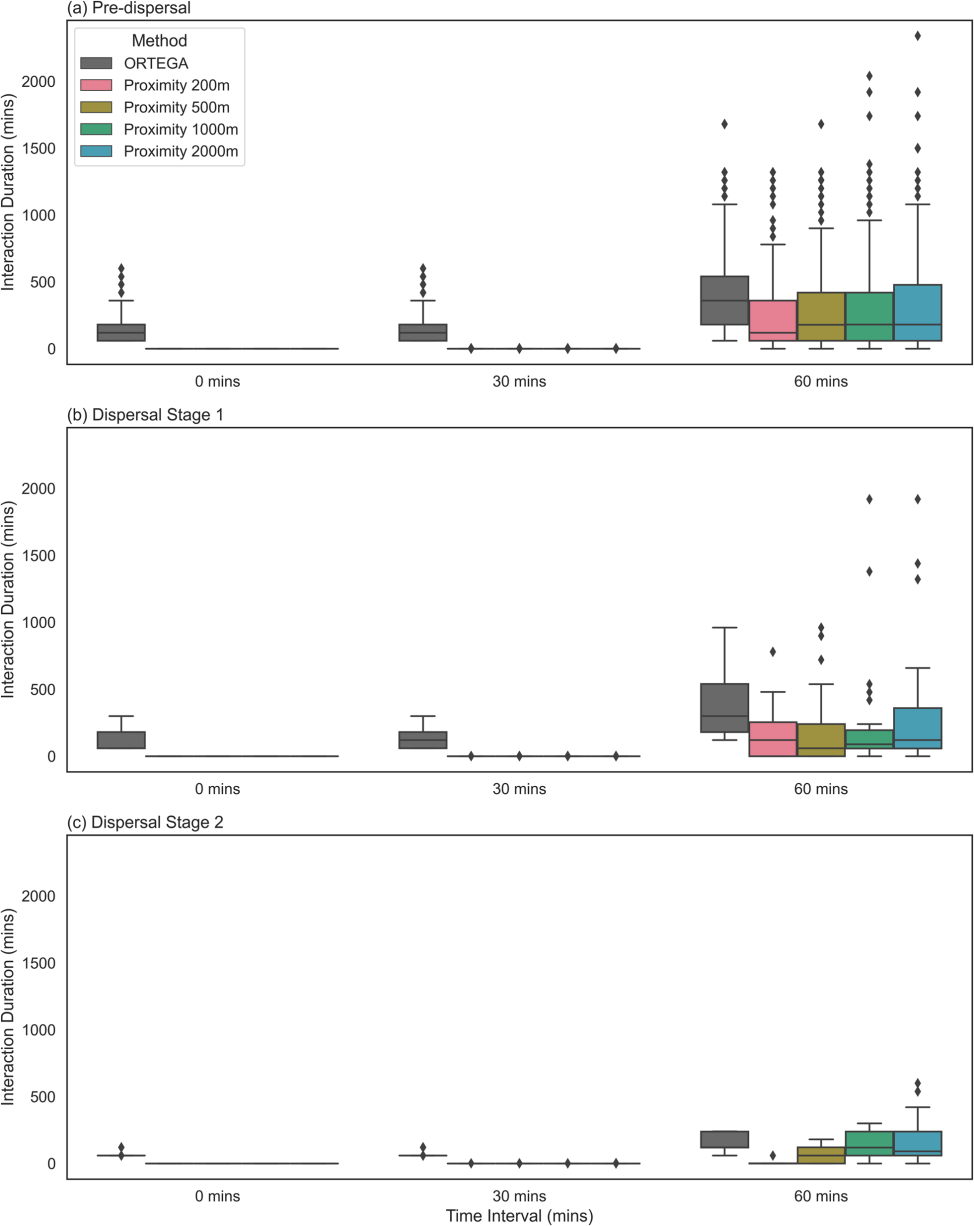
A comparative analysis of interaction duration for the mother tiger and young-2 as generated by ORTEGA and the proximity-based approach. Each subplot represents a stage, with interaction duration box-plotted at different time windows (0, 30, and 60 min) for the PPA or buffer intersections. Different colors distinguish between ORTEGA’s results in grey and proximity-based approach at various distances (200, 500, 1000, 2000 meter) in other colors. If a box plot does not appear for a particular method at a given time lag, it signifies that no interactions were detected using that method for the given time lag

Since the tracking data is collected at a 60-min interval, to detect concurrent interactions, we apply three different time windows (0, 30, and 60 min) in both ORTEGA and the proximity-based approach. Additionally, four spatial buffer thresholds (200, 500, 1000, and 2000 ms) are considered for the proximity-based approach. The results are visualized as box plots in Fig. 6. The Mann–Whitney U Test (summarized in Table 7) indicates that the median duration of interaction computed using the two methods are significantly different, especially in pre-dispersal and dispersal Stage 1.

The results suggest that ORTEGA better captures the potential interaction using PPA intersection, particularly noticeable during the pre-dispersal stage when the young stays together with the mother tiger. Conversely, the proximity-based approach fails to detect potential concurrent interactions using buffer intersection at this stage, especially when the time window is shorter than the temporal resolution of data, irrespective of the size of spatial buffers.

ORTEGA does not rely on a spatial buffer threshold. Therefore, its performance remains consistent. In contrast, the results of the proximity-based approach vary based on the size of the spatial buffer. In the pre-dispersal and dispersal Stage 1, the proximity-based approach captures more interactions when the buffer increases, with overall performance aligning with the results from ORTEGA results at the time window of 60 min. Moreover, the results of the proximity-based approach seems to overestimate the number of interactions especially with larger buffer sizes and temporal windows.

These experiments highlight the dependency of the proximity-based approach on simultaneous tracking and regular location updates to determine when individuals are in close proximity. As a result, larger time windows and spatial buffers may overestimate potential interactions, while smaller ones could underestimate them. That is, when tracking data are coarse or include gaps and the two animals are not observed at the same time, the proximity-based approach misses potential encounters. In contrast, since ORTEGA uses the PPA to consider the potential areas between the tracking points, it can better capture potential encounters in such cases. Furthermore, ORTEGA is capable of analyzing delayed interactions, while the common proximity-based approaches do not support this function.

### Mother–young interaction

#### Mother tiger and young-1

**Fig. 7:**
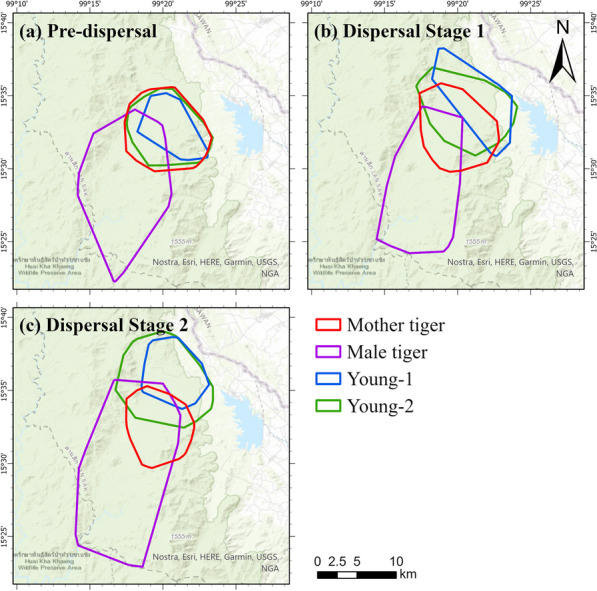
Shifts in home ranges of the mother tiger (in red), young-1 (in blue), young-2 (in green), male tiger (in purple) as the young disperse

**Fig. 8:**
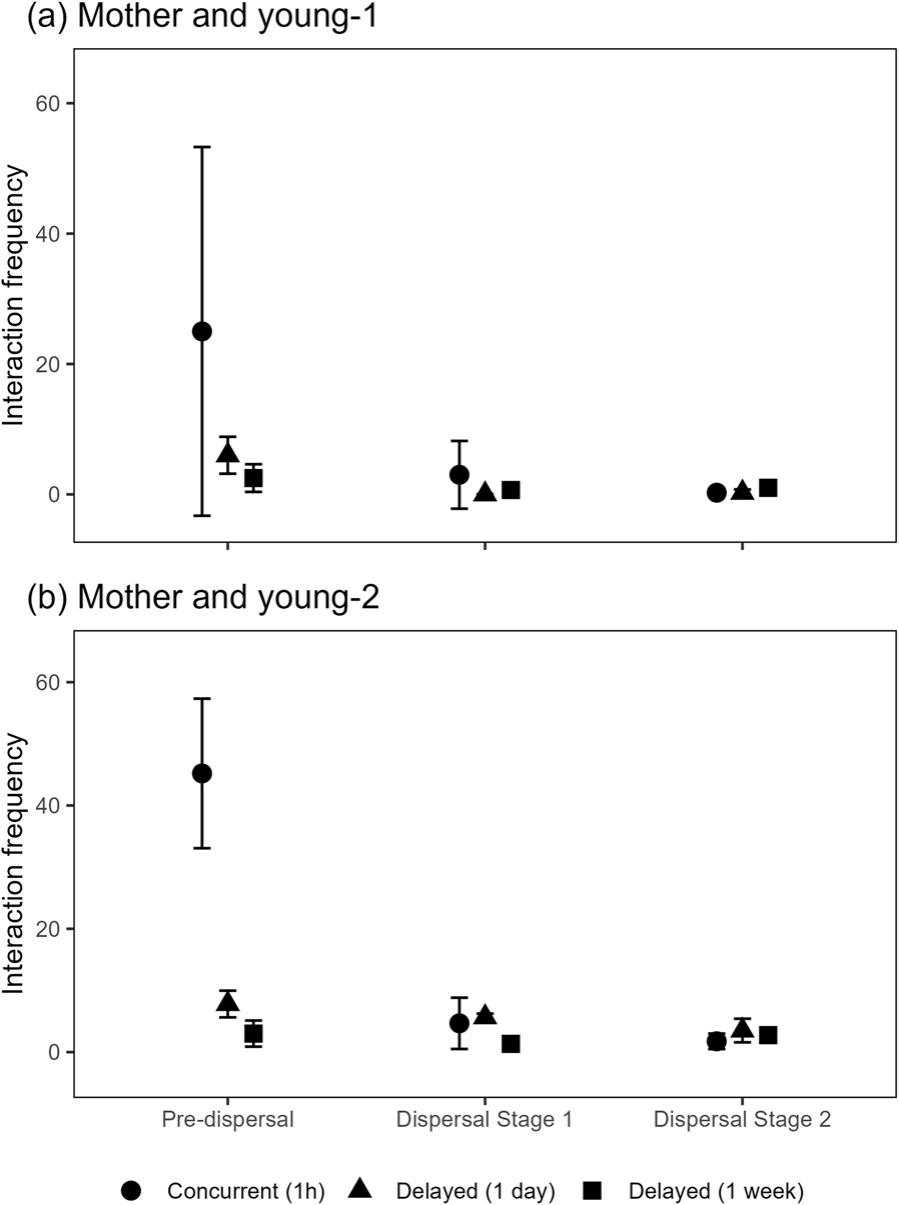
Frequency of concurrent and delayed interaction (for time lags of 1 day and 1 week) between mother tiger and **a** young-1 and **b** young-2 in pre-dispersal, dispersal Stage 1, and dispersal Stage 2. These frequencies represent how many times tigers come into a potential contact regardless of the duration of interaction

The outcomes suggest that young-1 maintains a close relationship with his mother before dispersal, as indicated by the overlapping red and blue home ranges in Fig. 7a and the concurrent interactions captured in Fig. 8a during the pre-dispersal phase. The home range area of young-1 during the pre-dispersal phase is 43.7 km^2^ (Table 3), with an overlap of 43.5 km^2^ with the mother tiger, accounting for 99.6% of the young-1’s home range (Table 1). During this phase, the number of observed incidences of interactions between young-1 and his mother per month are: $25 \pm 28.3$ incidences per month concurrently, $6.0 \pm 2.8$ incidences per month with a lag of 1 day, and $2.5 \pm 2.1$ incidences per month with a lag of 1 week. The longest monthly concurrent duration between the mother tiger and young-1 lasts around 256 h in January 2020.

As young-1 matures, he moves northeastward. His home range overlap with his mother decrease to 35.2% during Stage 1 of dispersal, and further decreases to 10.0% during Stage 2 of dispersal (Table 1). Only one concurrent interaction between the mother tiger and young-1 is detected in Stage 2 of dispersal in June 2020 (Fig. 8a). The results indicate a low level of interaction between these two tigers during dispersal.

#### Mother tiger and young-2

Compared to young-1, young-2 shows a closer relationship with their mother, with a larger shared home range and a higher frequency of interactions before and during dispersal. The home range overlap between young-2 and the mother tiger is 99.6% of young-2’s home range (Table 1). In terms of monthly interaction, young-2 and the mother show $45.2 \pm 12.1$ incidences of concurrent interactions before dispersal, $4.7 \pm 4.2$ incidences with a time lag of 1 day, and $1.8 \pm 1.3$ incidences per month with a time lag of 1 week (Fig. 8b). The monthly concurrent duration between the mother tiger and young-2 lasts 165 h in September 2019, 398 h in October 2019, 304 h in November 2019, 320 h in December 2019, and 334 h in January 2020 (Fig. 5). This indicates that young-2 stays close to the mother or follows her path and becomes independent of its mother more gradually.

The home range overlap between mother tiger and young-2 decreases to 61.0% during dispersal, and continued to decline to 26.2% in Stage 2 of dispersal (Table 1). Although there is a larger home range overlap, the frequency of concurrent interactions between the mother and young-2 drops to the similar level as young-1. A total of 14 concurrent interactions are detected during Stage 1 of dispersal, and 7 incidences are captured during Stage 2 of dispersal. The detected delayed interaction with a time lag of 1 day decreases from $5.7 \pm 0.6$ incidences per month during Stage 1 of dispersal to $3.5 \pm 1.9$ incidences per month during Stage 2 of dispersal (Fig. 8b). These results may indicate the awareness of the two tigers of the presence of one another, but less desire for concurrent interactions.

### Female–male interaction between the mother tiger and the male tiger

**Fig. 9:**
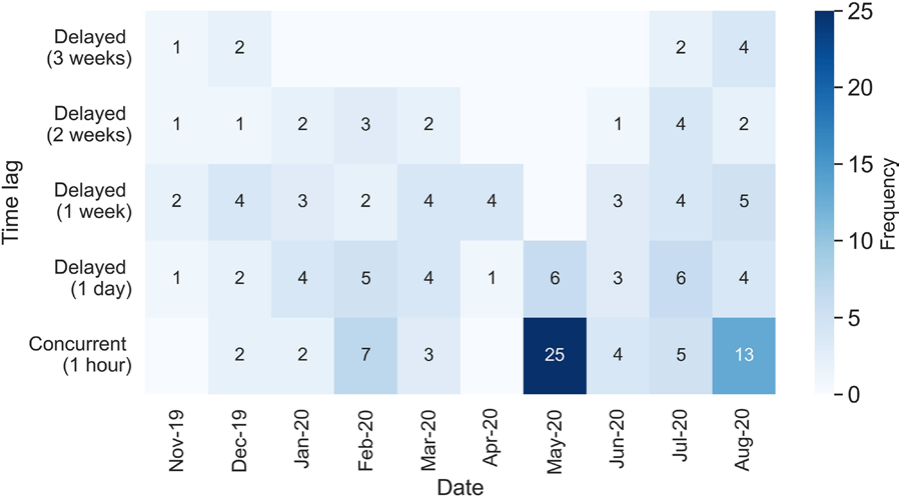
A heat map of representing the frequencies of concurrent and delayed interactions between the mother tiger and the male tiger in each month at a time lag from 1 day to 3 weeks. Darker colors represent higher interaction frequencies

Figure 9 summarizes the outcomes of interaction analysis between the male tiger and the mother tiger. The results indicate a higher level of interaction between the dyad in the month of May 2020, after the young’s dispersal. Although the home range of the male tiger covers 34.9% of the home range of the mother tiger before the young disperse (Table 1), the male tiger tends to avoid concurrent interactions with the mother tiger when the young are still in a close relationship with their mother (November 2019–Jan 2020). The young start to disperse when the male begins to interact with the mother. During Stage 1 of dispersal, he had 10 concurrent interaction and 10 delayed interactions at a time lag of 1 week with the mother tiger (Fig. 9). The proportion of home range overlap increases to 40.5% during dispersal of the young. During Stage 2 of dispersal, the coverage increases to 82.9%, which indicated that the male tiger home range includes most of the female tiger home range (purple-red dyad in Fig. 7c). The longest concurrent interaction between the male tiger and the mother tiger lasts 25 h from May 29 17:00 to May 30 18:00 (Table 6). The average frequency of the two dyad’s concurrent interaction after May 2020 decreases to 7.3 times per month on average, with an average duration of 31 h each month (Fig. 9).

As mature tigers, both the mother and the male tiger have stable home ranges. The mother tiger’s home range of $67.8 \pm 14.9$ km^2^ is nearly round-shape, while the male tiger maintains a larger home range of $143.6 \pm 30.5$ km^2^, around 2–3 times the size of the mother tiger’s. The mother tiger shifts her western home range boundary toward the east after the young dispersal, as a result, more space is available to the neighbor tiger (red polygons in Fig. 7). This suggests that the male tiger avoids visiting the shared home range with the mother tiger prior to the young dispersal, and patrols more often post-dispersal.

### Male–male interaction

**Fig. 10:**
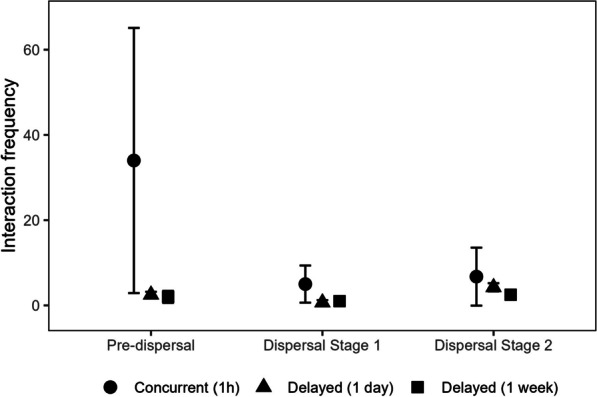
The frequency of concurrent and delayed (time lags of 1 day and 1 week) interactions between young-1 and young-2 during the pre-dispersal, dispersal Stage 1, and dispersal Stage 2

**Fig. 11:**
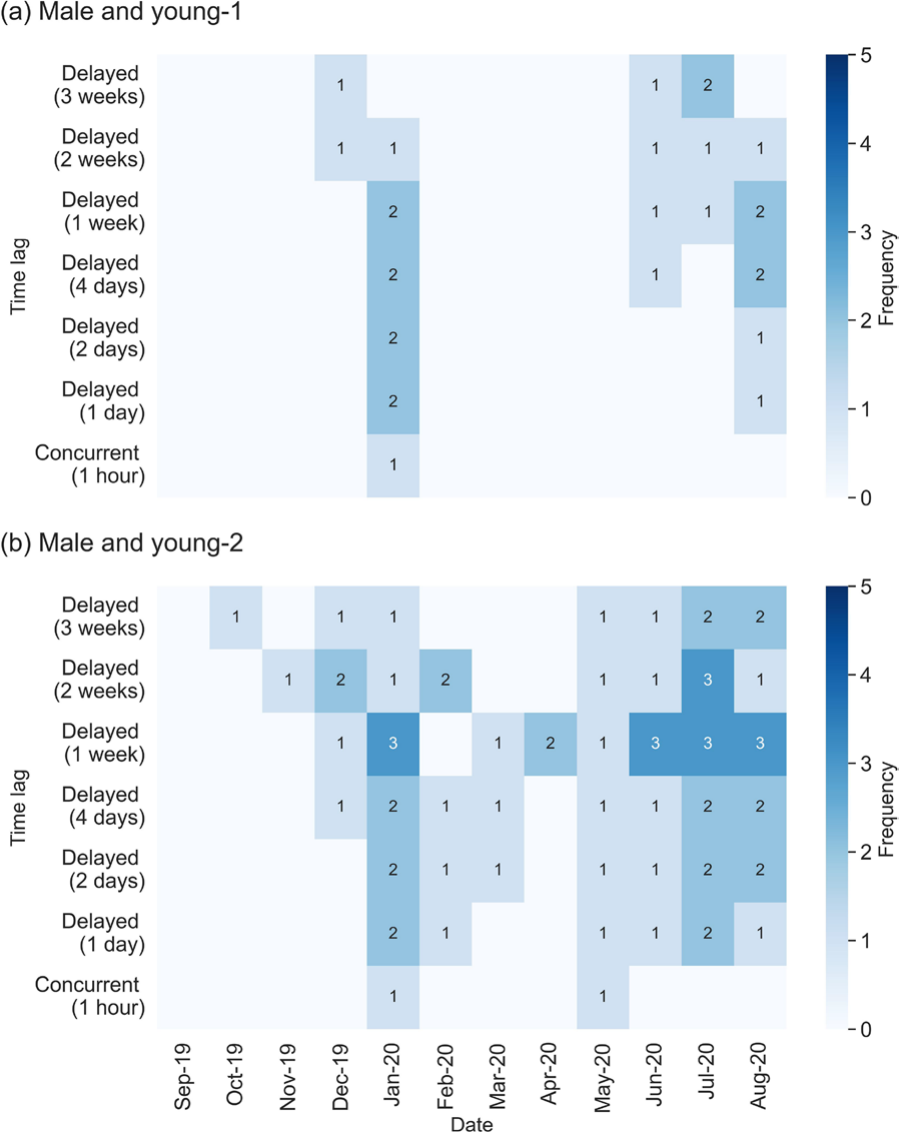
A heat map representing the monthly frequency of concurrent and delayed interactions between the adult male tiger and **a** young-1, and **b** young-2 for a time lag from 1 day to 3 weeks. Darker colors represent higher interaction frequencies

We explore two types of male–male tiger interaction in this section: between subadult males (Fig. 10) and between subadults and adult males (Fig. 11).

#### Young-1 and young-2

Figure 10 suggests that the two young stay close together (concurrent interaction = 34.0 ± 31.1 per month with an average duration of 8.2 h) or follow each other (delayed interaction counts with a delay of 1 day = 5.0 ± 4.4 per month) before dispersal. In February 2020, the two young male tigers move north to establish their own territories, sharing the same territory but staying separated until April 2020 (Fig. 7). In the following 4 months (dispersal Stage 2), their interaction drops to $6.8 \pm 6.8$ incidences per month concurrently with an average duration of 4.0 h. Their concurrent interaction frequency reaches a maximum of 16 incidences in June 2020 with a duration of 67 h. Their delayed interactions over a time lag of 1 day are $4.3 \pm 1.0$ incidents per month during Stage 2 of dispersal and it reduces to no interaction after July 2020.

Both young male tigers shift their home ranges toward north, but still share a portion of their mother’s territory during Stage 1 of dispersal. By Stage 2 of dispersal the young males seem to try to establish their territories. Notably, young-2 shows more movement compared to young-1. Table 3 shows that the home range area of young-2 is larger than young-1 at all stages, which may be reflective of its more active movement patterns.

#### Male tiger and two young

The results underline the tendency of male tigers to avoid interaction, which is reflected in the smaller overlap in home range between the three male tigers compared to the overlap between the mother tiger and the adult male tigers. Prior to the young male’s dispersal, the home range overlap areas between the adult male and the two subadult tigers are 8.4 km^2^ and 23.7 km^2^, respectively (Tables 1 and 3). However, during their dispersal, the coverage decreases to none for the young-1 and 13.8 km^2^ for the young-2 (blue-purple dyad in Fig. 7b, Table 1). The adult male tiger starts to interact more with the mother tiger once the young leave her (Fig. 9).

The results suggest fewer concurrent interaction or delayed interactions with a time lag shorter than 1 week between the male tiger and the young, although their home range has a small overlap before the young dispersal. The number of first delayed interactions across different time lags (1 day to 3 weeks) is shown in Fig. 11. No interaction is detected between the male and young-1 during dispersal Stage 1.

With the increase in time lag, more delayed interactions are detected between young-2 and the male tiger during stage of dispersal. Most male–male delayed interactions are detected in July 2020 when young-2 moves back to the southern area of the home range where the adult male is. However, the interaction with young-1 stay minimal (average of 1–2 incidences per month) as he does not return to the southern area.

These results may indicate that although the male tigers avoid encounters, they might have an awareness of one another, as they tend to check the visited locations of the other tiger with a delay of about 1 day up to 3 weeks. Delayed interactions after 3 weeks might be more incidental, as the field observations suggest that the scent marks can last about up to 3 weeks [24].

### Female–female interaction between the mother and the neighbor female tiger

**Fig. 12:**
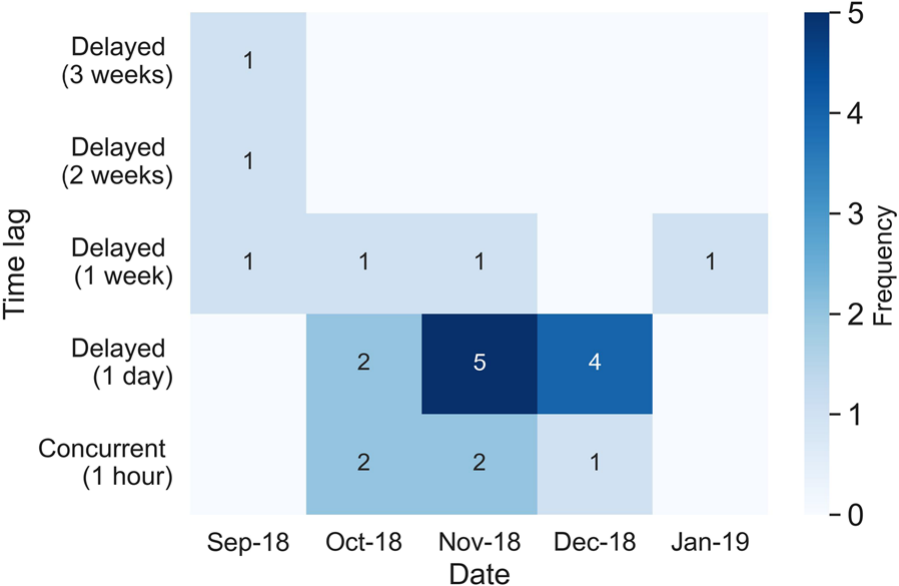
A heat map representing the monthly frequency of concurrent and delayed interactions between the mother tiger and the neighbor female tiger in each month at a time lag from 1 day to 3 weeks. Darker colors represent higher interaction frequencies

The interaction analysis results suggest a relatively low frequency and duration of interaction between the two adult female tigers. During the 5 months tracking period, only two concurrent interactions are detected in October 2018, two incidents in Nov 2018, and one incident in December 2018 (Fig. 12), with the total duration of concurrent interaction of 12 h.

**Fig. 13:**
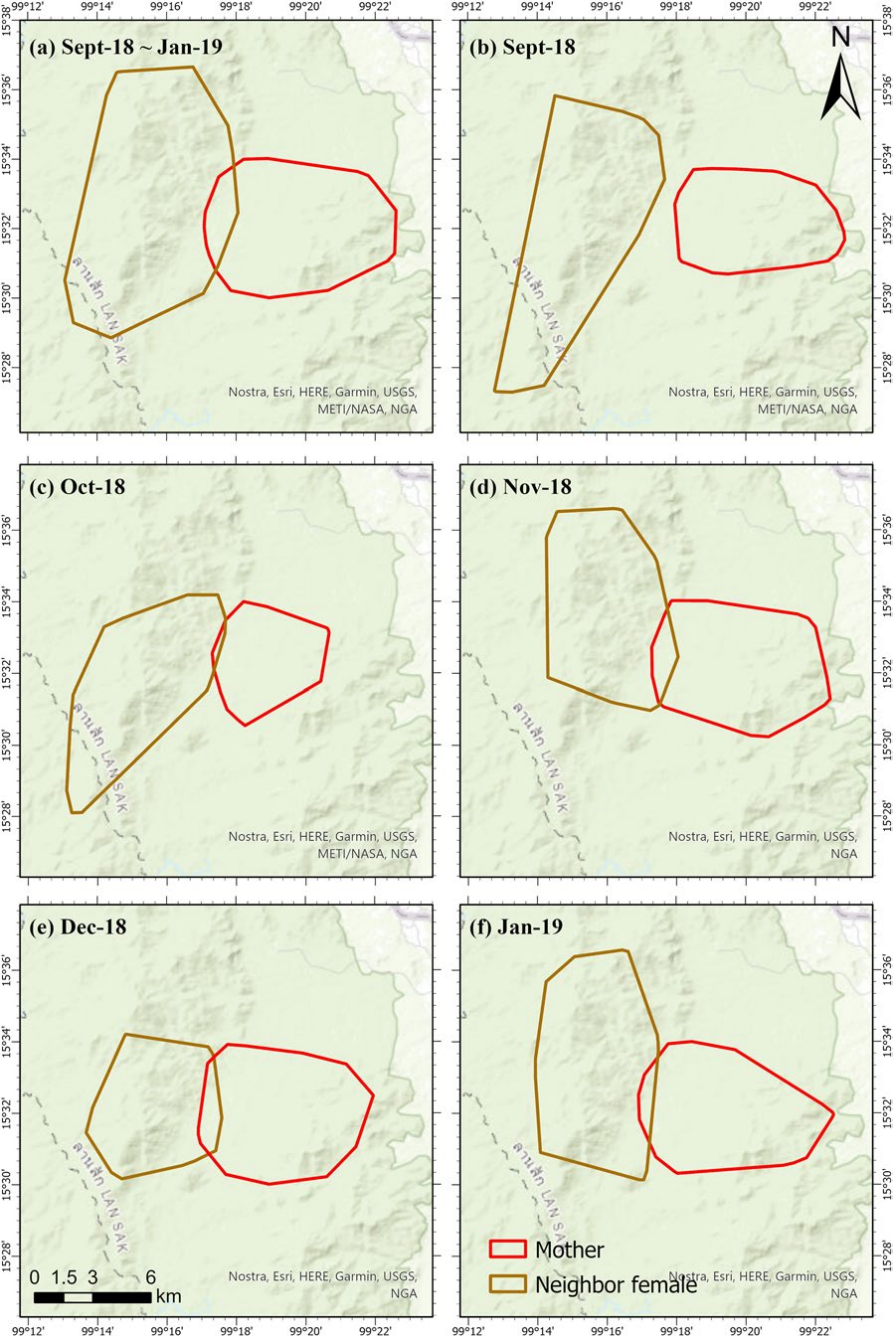
Shifts in home ranges of the mother tiger (in red) and the neighbor female tiger (in brown) in 5 months

Figure 13 and Table 2 demonstrate a small shift in the home ranges of the two tigers at the shared boundary over time. In September 2018, no overlap exists between the home ranges (95% tracking points) of the two female tigers, leading to no concurrent interaction. One delayed interaction with a time lag of 1 week can be detected within the remaining 5% tracking points. During October 2018 and January 2019, the monthly concurrent interaction between the two animals stays low (less than two incidences). Their home range overlaps varies from 0.6 km^2^ in October to around 3.8 km^2^ in November and December, and 2.3 km^2^ in January. The detected monthly delayed interaction is relatively low, at a rate of two to five visits to the same location at a time lag of 1 day to 1 week. These results may indicate that the two tigers effectively established adjacent territories; they avoid encountering one another and scent mark their common boundary less often.

## Discussions

### Behavioral patterns and impact of interaction

Based on the home range and interaction dynamics of the five tigers, we discuss four types of behavioral patterns occurring in tiger intraspecific interaction: following, encounter, latency, and avoidance.

#### Following

**Fig. 14:**
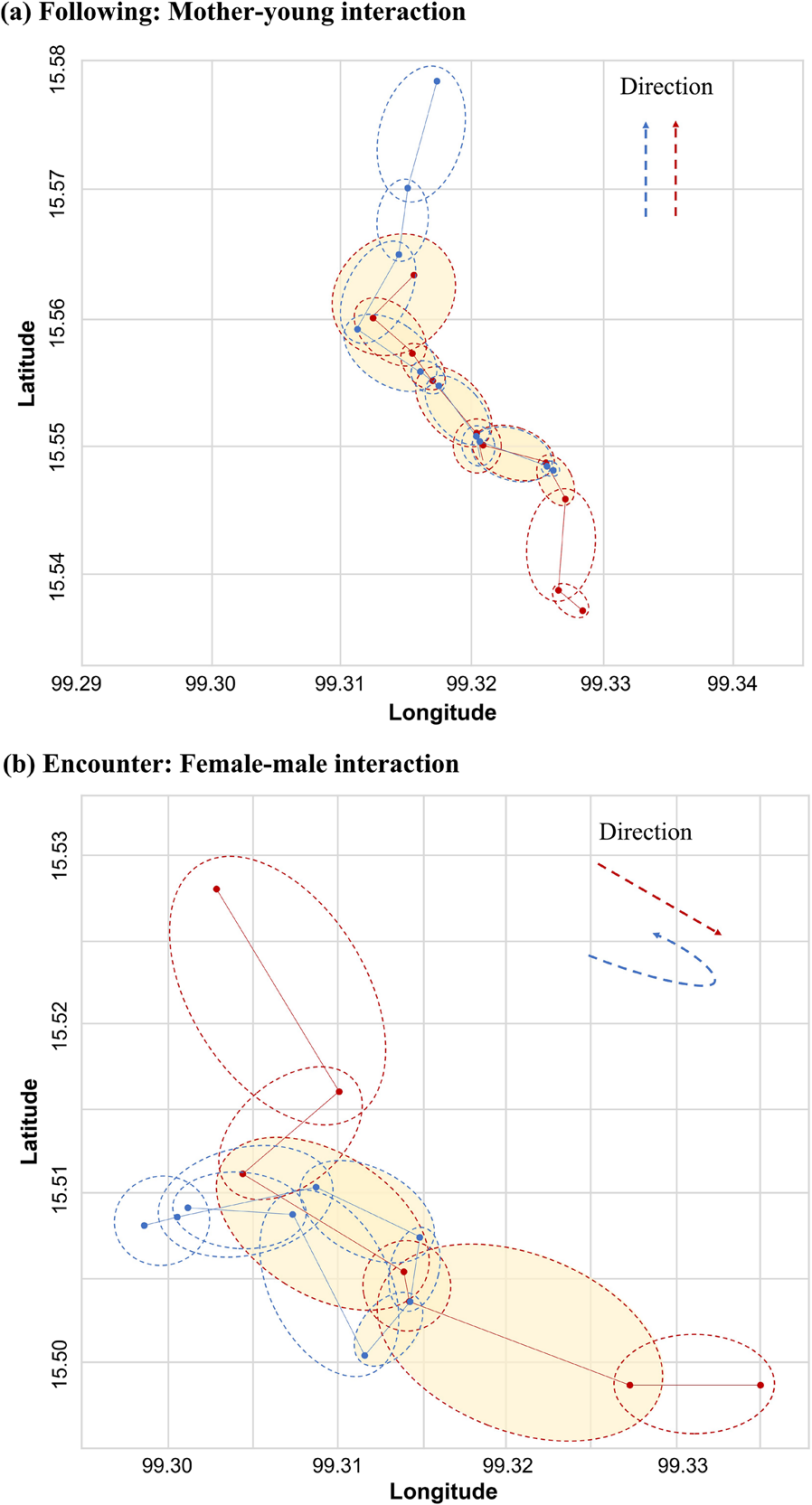
A schematic representation of a continuous interaction segment between **a** mother–young (duration: 7 h) and **b** female–male (duration: 3 h). For visual clarity, the location of stops are removed. Light yellow shaded ellipses highlight the intersecting PPAs. Directions of two trajectories are shown in the top-right corner of each sub-figure

**Fig. 15:**
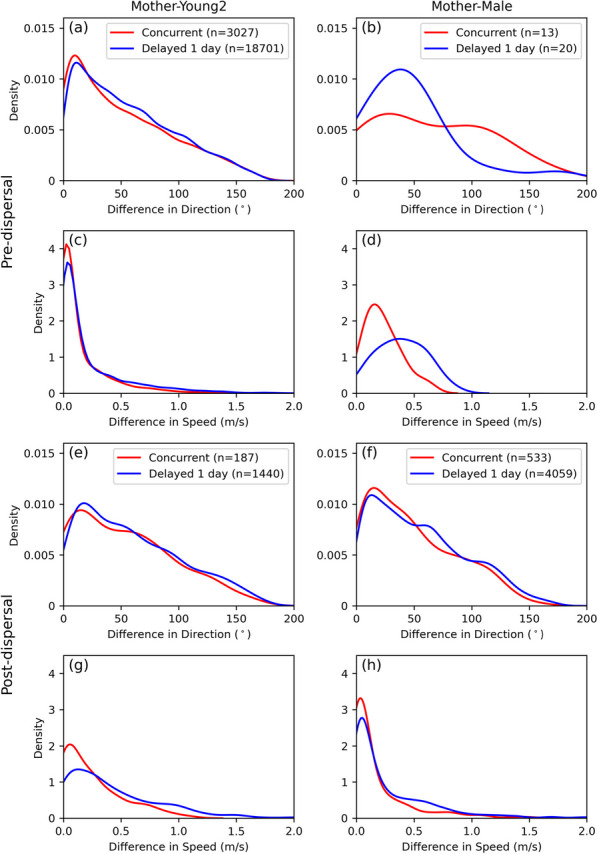
Kernel density estimations of two movement parameters (difference in movement direction and speed) for two pairs (Mother–Young2, Mother–Male) of intersecting PPAs during concurrent and delayed interactions. The differences in direction and speed are calculated as the absolute value. The first two rows correspond to the pre-dispersal period, and the third and fourth rows represent the post-dispersal period (encompassing dispersal Stage 1 and 2). Each column corresponds to a different pair. Within the subplots, concurrent interactions are denoted in red, delayed interactions with a 1-day lag are in blue. Parenthetical numbers in the legends are the total number of intersecting PPA pairs contributing to each line

A “following” behavior occurs when two moving entities move in the same direction, either simultaneously or with a time lag. This is characterized by parallel movement direction and similar speed between the interacting dyads (Fig. 14a). This pattern is commonly observed in mother–young interactions prior to dispersal when the young followed the mother (e.g. between mother and Young2 in Fig. 15a, c) or between the male tiger and the mother tiger after dispersal of her youngs (Fig. 15f, h). This can also be observed when the young start to disperse. They divide their time between exploring the habitat semi-independently both within and adjacent to their natal area and then exhibit concurrent or delayed following behavior with their mother. Although these interactions occur by a lower frequencies, they maintain a similar movement direction and speed as shown in Fig. 15e and g.

#### Encounter

An “encounter” occurs when two animals move from different directions and meet at the same location for a short period of time (Fig. 14b). This interaction type can be quantified by a higher difference in movement direction in concurrent interactions. It is particularly observed during female–male concurrent interactions on a monthly basis prior to the dispersal of young, as observed in the sparse distribution of their random movement direction (Fig. 15b) and speed (Fig. 15d). The male tiger meets the female tiger on patrol, then changes direction and moves with the female tiger for a short period of time and then moves on. This type of pattern is detected as regular periodic “encounters” in tracking data of the male and female tigers and provide insights into the territorial and mating behaviors of the two tigers.

#### Latency

A “latency” pattern is observed when a tiger indirectly communicates and responds to another’s previous behavior with a time lag. It often happens when a tiger enters another tiger’s home range or when the two female tigers patrol their common territory boundary. In the female–female interaction we analyzed, a “latency” pattern is observed starting with no encounters and little to no delayed interaction between them, then the frequency of delayed interaction gradually increases (Fig. 12). Eventually, the intensity of interaction increases resulting in some concurrent encounters which led to a shift in their home ranges (Fig. 13). Then behavior shifts to “avoidance”. “Latency” is also a notable pattern in female–male interactions (Fig. 9), especially related to mating behavior, when a male periodically visits a female’s territory to check her reproductive status. When the cubs are 15–18 months old the mother often comes into pre-estrous and her scent marks indicate the approach of estrous [24].

#### Avoidance

**Fig. 16:**
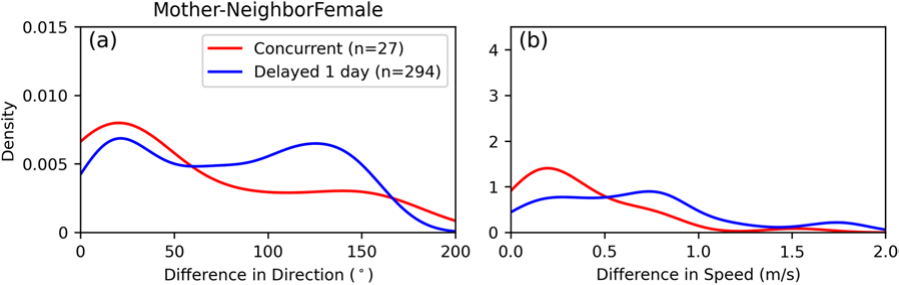
Kernel density estimations of difference in **a** movement direction and **b** speed for Mother–Neighbor Female at intersecting PPAs during concurrent (shown in red) and delayed interactions with a 1-day lag (in blue). Parenthetical numbers in the legends are the total number of intersecting PPA pairs contributing to each line

**Fig. 17:**
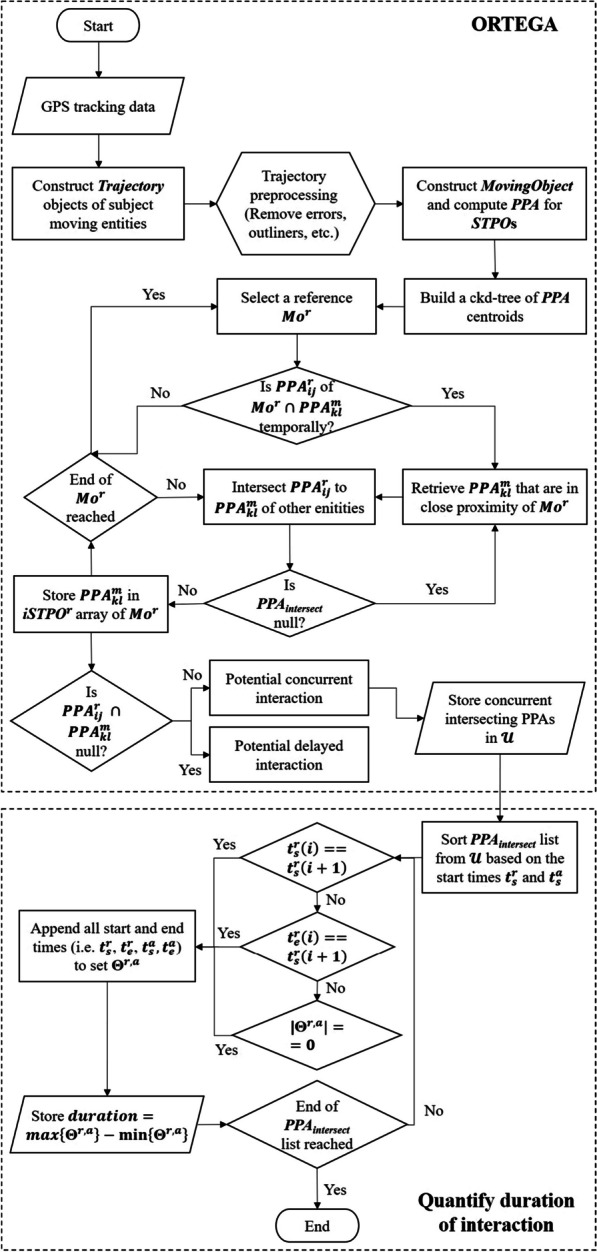
Workflow of identifying and quantifying the duration of concurrent and delayed interactions (modified from [31, 38])

“Avoidance” happens when a tiger alters its behavior or path upon detecting another’s presence, often through scent marks of a neighbor. This is the dominant behavior pattern in both female–female and male–male interactions. These delayed interactions along a common territorial boundary allows females to establish territorial boundaries while avoiding aggressive encounters, and thus mitigating the probability of getting injured. This behavioral pattern is characterized by a low density and variable differences in movement direction (Fig. 16a) and speed (Fig. 16b) Avoidance is also common between adjacent males until a female comes into estrous near a territorial boundary. Then the behavior shifts to “encounter”, which often leads to injuries in which a resident male ejects or is ejected by a rival male.

### Speculation of tiger biological state and impact on interaction

#### Association of mother and resident male in May 2020

When the female comes into estrous scent marking decreases and the female begins to repeatedly call 20 to 40 times, and the male often calls back with the result of an encounter that leads to a *following* behavior in the form of an extended association and mating [24].

We speculate from the behavioral patterns and interactions captured in the data that the male mating with the resident female during an extended period of repeated “following” behavior from May 23 to May 30, 2020. Tigers are induced ovulators [69, 70], and continuous following behavior typically occurs when male and female tigers associate for several days. Smith and McDougal [71] observed similar following data and observed a mating association that lasted several days and predicted several other successful mating events. Our analysis of movement data provides a means to remotely identify these mating associations.

#### New litter during March–April 2020

We also speculate that the mother tiger probably has a new litter resulting in the dispersal of her young. Newborn cubs are confined to the den and the female tiger’s activity is restricted close to the den sites for the first 2 months [29]. Subadult tigers become semi-independent when their mother gives birth to the next litter, but they remain within their mother’s territory until the new litter starts to move with their mother [26]. By conducting trajectory segmentation, we find that the hunting duration and frequency of the mother tiger from March to April 2020 is only 30% of the average value, which also matched the start time of the young dispersal. Moreover, there is no concurrent or delayed interaction detected between the mother and the maturing young during that period, but they benefit by hunting in their natal area, and in doing so, they help maintain the mother’s territory while her movements are confined. Their dispersal may be precipitated and begins to expand the use of her territory as a new litter becomes more mobile. After the duration of patrolling of the mother tiger increases to the average value in May 2020, an encounter between the mother tiger and young-2 is detected at the end of May. More delayed interaction between the mother and the dispersing offspring is observed later.

### Limitations and future work

The interaction analysis approach utilized in this paper, ORTEGA, uses time geography as a deterministic approach to estimate activity space, where the entire intersecting PPAs are considered as areas for potential interactions regardless of the size of intersection areas. Building on the foundational work by Downs et al. [61], Winter and Yin [57], and Song and Miller [56], future work can focus on developing a probabilistic time geography method by integrating ecological movement models to model interaction probabilities within the space–time prisms. These methods encompassing voxel-based representations, directed random walks, and truncated Brownian Bridges, offer a more dynamic approach to capturing the likelihood of interactions at the intersected PPAs by considering visit probabilities within the prisms. Integrating ORTEGA with probabilistic approaches, such as Brownian bridges and utilization distribution [55] can help generate insights into how animals utilize space and time, offering a complementary perspective to the PPA intersections and enhancing the precision and applicability of ORTEGA in movement ecology.

ORTEGA is a generic method that is adaptable to a wide range of species for tracing concurrent and delayed interactions in animal tracing data, but its application must account for scale effects and temporal sampling rates as the estimation of contacts can be impacted by the observation scale. For example, As shown in [65], with coarser tracking data, while the proximity based approach becomes almost invalid, ORTEGA might slightly overestimate the number and duration of interactions. Therefore, depending on the movement ranges of the subject animals, having higher resolution data of 20 min to 1 h would be more beneficial. As such, adjustments in spatial and temporal resolutions are necessary to accommodate species diversity. Specifically, smaller, faster-moving species might require finer temporal sampling, whereas larger species may need a broader spatial scale for analysis. The tiger data presented in this case study is captured at an 1-h interval.

Despite ORTEGA’s advanced approach, it is worth noting that the proximity-based approach remains a useful tool for detecting concurrent interactions, especially with regular, high-resolution tracking data. When the temporal and spatial resolutions are adequately dense, proximity-based methods can provide valuable insights into the spatial dynamics of animal interactions. Such insights complement ORTEGA’s comprehensive analysis, highlighting the need for flexible, context-sensitive approaches in studying animal movements.

The discussion of the different types of interactions in this study is supported by domain expert knowledge and field observations of tiger behavior. Future research should consider incorporating additional environmental and behavioral information to identify higher levels of interaction patterns, such as “conflict” and “mating”, and how animals interact with the physical and social environment [72] through context-aware interaction analysis. Moreover, there is a high degree of overlap between tigers and leopards in Huai Kha Khaeng, but elsewhere across their range interference competition results in strong leopard avoidance of tigers. The analysis presented here may aid future research on how spatial and movement dynamics influence interactions of tiger and leopards and other carnivore guilds. Future research should also focus on investigating and contextualizing more complex delayed interactions between tigers (e.g. scent marking) and whether leopards overlapping with tigers avoid temporal interference competition.

## Conclusions

This paper presented the applicability of time-geography in animal movement behavior analysis. The proposed interaction analysis method, ORTEGA, provide promising opportunities to apply the time geography theory in analysis and understanding of higher-level behavior of wildlife, such as predator–prey dynamics, competition interference, and interspecific and intraspecific interactions in carnivore research. Specifically, this paper presented a case study of analyzing spatio-temporal interaction patterns of multiple tigers over several months through GPS tracking. We showed the shifts in spatial and temporal interaction patterns in two adult female, one adult male, and two male young tigers and demonstrated how interactions impacted their home ranges. We described four primary types of interaction patterns (encounter, following, latency, avoidance) that can be captured based on home range dynamics and spatial and temporal characteristics of PPA intersections using trajectory data. Furthermore, we assessed the performance of our proposed technique, ORTEGA, in comparison to the proximity-based approach in interaction analysis. We showed that ORTEGA more robustly captures concurrent interaction, while the proximity-based results vary by the size of spatial and temporal buffers.

## Data Availability

The python package of ORTEGA algorithm and example codes are uploaded on GitHub https://github.com/move-ucsb/ORTEGA. The GPS tracking data cannot be published publicly due to the sensitive nature of tiger locations.

## Appendix

See Tables 3, 4, 5, 6, 7 and Fig. 17.

**Table 3:** Summary of home range areas (km^2^) of mother tiger, male tiger, young-1, and young-2 at three stages in their gradual independence from their mother

Tiger	Pre-dispersal	Dispersal	
		Stage 1	Stage 2
Mother	86.1	80.8	64.0
Male	163.5	142.7	217.5
Young-1	43.7	68.5	53.0
Young-2	73.9	89.3	103.5

**Table 4:** Summary of home range areas (km^2^) of the mother tiger and the neighbor female tiger in 5 months

Tiger	Sept-18	Oct-18	Nov-18	Dec-18	Jan-19
Mother	40.2	26.1	50.6	50.5	49.1
Neighbor female	74.0	52.5	55.9	39.3	61.0

**Table 5:** Summary of the frequency of concurrent and delayed interaction among mother and two young tigers during pre-dispersal, dispersal Stage 1, and dispersal Stage 2

Dyad	Interaction	Pre-dispersal	Dispersal	
			Stage 1	Stage 2
Mother–young-1	Concurrent (1 h)	25.0 ± 28.3	3.0 ± 5.2	0.3 ± 0.5
	Delayed (1 day)	6.0 ± 2.8	0.0	0.3 ± 0.5
	Delayed (1 week)	2.5 ± 2.1	0.7 ± 1.1	1.0 ± 0.8
Mother–young-2	Concurrent (1h)	45.2 ± 12.1	4.7 ± 4.2	1.8 ± 1.3
	Delayed (1 day)	7.8 ± 2.2	5.7 ± 0.6	3.5 ± 1.9
	Delayed (1 week)	3.0 ± 2.1	1.3 ± 1.2	2.8 ± 1.0
Young-1–young-2	Concurrent (1h)	34.0 ± 31.1	5.0 ± 4.4	6.8 ± 6.8
	Delayed (1 day)	2.5 ± 0.7	0.7 ± 0.6	4.3 ± 1.0
	Delayed (1 week)	2.0 ± 1.4	1.0 ± 1.0	2.5 ± 1.0

**Table 6:** Summary of the minimum concurrent interaction duration per day and per month between mother and male tigers in 2019 and 2020

Year	Date	Start time	End time	Duration/event (h)	Duration/month (h)
2019	Dec-29	18:01	20:00	2	3
	Dec-30	5:00	6:00	1	
2020	Jan-9/10	23:01	2:00	3	6
	Jan-29	4:00	7:00	3	
	Feb-10/11	22:00	3:00	5	24
	Feb-11	6:00	8:00	2	
	Feb-12/13	20:00	3:00	7	
	Feb-14	20:00	23:00	3	
	Feb-15	13:00	18:00	5	
	Feb-17	3:00	5:00	2	
	Feb-29/Mar-1	23:00	10:00	11	29
	Mar-1	12:00	18:00	6	
	Mar-1/2	19:02	4:00	9	
	Mar-2	5:02	8:00	3	
	May-1/2	23:00	3:00	4	147
	May-2	4:00	11:00	7	
	May-2	14:00	16:00	2	
	May-2	18:00	21:00	3	
	May-4	20:00	22:00	2	
	May-17	6:00	7:00	1	
	May-17/18	23:01	5:00	6	
	May-19	5:00	8:00	3	
	May-22	10:00	16:00	6	
	May-22/23	16:01	1:00	9	
	May-23	4:00	8:00	4	
	May-23	9:00	15:00	6	
	May-23	16:01	21:00	5	
	May-23	22:00	23:00	1	
	May-24	1:00	8:00	7	
	May-24/25	20:00	2:00	6	
	May-25	4:00	10:00	6	
	May-25	11:00	15:00	4	
	May-25/26	16:01	7:00	15	
	May-27	19:00	23:00	4	
	May-28	2:01	11:00	9	
	May-28	15:01	18:00	3	
	May-28	19:01	21:00	2	
	May-28/29	21:01	4:00	7	
	May-29/30	17:00	18:00	25	
	Jun-6	2:01	5:00	3	24
	Jun-26	18:00	22:00	4	
	Jun-27	1:00	7:00	6	
	Jun-27	8:00	19:00	11	
	Jul-21	5:00	6:00	1	13
	Jul-26	17:00	21:00	4	
	Jul-28	6:00	9:00	3	
	Jul-28	19:00	21:00	2	
	Jul-29	5:01	8:00	3	
	Aug-4/5	23:00	9:00	10	56
	Aug-6/7	18:02	2:00	8	
	Aug-7	16:00	20:00	4	
	Aug-8	7:01	10:00	3	
	Aug-8	11:00	14:00	3	
	Aug-8/9	18:00	0:00	6	
	Aug-9	4:00	9:00	5	
	Aug-9	10:00	14:00	4	
	Aug-9	17:00	19:00	2	
	Aug-9/10	23:00	1:00	2	
	Aug-10	4:00	8:00	4	
	Aug-12	2:00	5:00	3	
	Aug-22	7:00	9:00	2	

**Table 7:** Statistical summary of performing a Mann–Whitney U test between ORTEGA and the proximity-based approach at the time window of 60 min

Stage	Spatial threshold (m)	Critical value^a^
Pre-dispersal	200	52918.0***
	500	50006.0***
	1000	51752.0***
	2000	58180.0***
Dispersal stage 1	200	126.5**
	500	172.5**
	1000	262.5***
	2000	364.0**
Dispersal stage 2	200	35.0***
	500	50.0*
	1000	57.5
	2000	139.5

^a^***$$0<p<0.01$$; $$0.01 \le p < 0.05$$; *$$0.05 \le p < 0.1$$
